# Evaluating the Mechanical Properties of Zinc-Coated Stainless Steel Orthodontic Wires Using Physical Vapor Deposition

**DOI:** 10.1155/2021/6651289

**Published:** 2021-05-03

**Authors:** Maryam Karandish, Mahmoud Pakshir, Milad Moghimi, Dana Jafarpour

**Affiliations:** ^1^Dept. of Orthodontics, School of Dentistry, Shiraz University of Medical Sciences, Shiraz, Iran; ^2^Department of Materials Science and Engineering, School of Engineering, Shiraz University, Shiraz, Iran; ^3^Student Research Committee, School of Dentistry, Shiraz University of Medical Sciences, Shiraz, Iran; ^4^Biomaterials Research Center, School of Dentistry, Shiraz University of Medical Sciences, Shiraz, Iran

## Abstract

The aim of this study was to evaluate the mechanical properties of stainless steel (SS) orthodontic wires coated with zinc (Zn), using a Physical Vapored Deposition (PVD) machine. A total of 100 straight SS orthodontic wires were cut into pieces of 5 centimeters in length and were divided into two groups. Half of the wires were coated with Zn using a PVD machine, and the others remained uncoated. Tensile strength (*n* = 15), three-point bending (*n* = 15), and frictional resistance at 0° (*n* = 10) and 10° (*n* = 10) were measured to compare the mechanical properties of the Zn-coated and uncoated orthodontic wires using the universal testing machine. The surface of the coated wires was observed by SEM and AFM. An independent *t*-test, multivariate ANOVA, and measurement ANOVA were used for data analysis. SEM and AFM showed a homogenous Zn layer of 0.28 ± 0.006 *µ*m on the SS wires. The tensile strength and three-point bending strength significantly increased after Zn coating of wires with the PVD method (*P* < 0.05). The friction resistance significantly reduced at both angulations following the coating procedure. The angle between the wire and bracket had no significant effect on the frictional resistance (*P* > 0.05). Coating with Zn improved the tensile and load-bending strength of SS orthodontic wires and reduced their frictional resistance which might be advantageous in terms of reducing the risk of root resorption during the orthodontic treatment.

## 1. Introduction

In recent years, new generations of wire alloys have been introduced in orthodontics. Stainless steel (SS) is widely used in orthodontics to manufacture brackets, archwires, bands, ligatures, and other appliances [1]. SS wires present high values in the modulus of elasticity and yield strength, high stiffness, environmental stability, and low cost [2, 3]. The development of these wires with enhanced mechanical properties has led to versatility of orthodontic treatment. Nevertheless, antibacterial performance and friction properties are among the features still required to be improved in order to optimize orthodontic treatment and enhance oral health [4–6].

The timely nature of orthodontic treatment would bring out concerns in the modification of the microbial environment and lead to increased proliferation of the bacterial population, which consequently results in a reduced pH [7, 8]. This might direct the demineralization-remineralization balance toward mineral loss, leading to the development of white spot lesions [9].

Due to medical advantages of Zn ion, it has been widely applied in medicine and dentistry. With regards to oral cavity, Zn compounds have exhibited numerous biological effects, such as reduced amount of oral bacteria, dental plaque, and dental caries [10, 11]. Moreover, the reduction in enamel demineralization rate was confirmed *in vitro* [12, 13]. Furthermore, Zn ions are assumed to increase subsurface remineralization in some circumstances, favoring great amounts of fluoride-induced surface mineralization [13, 14].

Furthermore, reduced frictional forces between wires and brackets have been previously reported which might contribute to a significant reduction in friction during tooth movement [15, 16].

While few studies measured the mechanical properties of SS wires coated with other materials, such as TiN and silver [17, 18], the effect of Zn coating on the improvement of mechanical properties, such as tensile strength, flexural strength, and friction resistance of NiTi wires has been supported [11].

Physical Vapored Deposition (PVD) coating, as an ecofriendly procedure, can reduce toxic elements that have to be discharged with more conventional types of coatings, such as fluid precursors and chemical reactions.

Due to the antibacterial effect of Zn coatings on orthodontic wires, this option could favor the clinician if it does not affect the mechanical properties of the wires. There are few investigations on the mechanical properties of coated SS wires, and to the best of our knowledge, no research has been conducted to evaluate these properties of Zn-coated SS wires by means of the PVD method.

## 2. Materials and Methods

This study was approved by the institutional ethical committee (IR.SUMS.DENTAL.REC.1398.008). A total of 100, 0.019 × 0.025 straight SS orthodontic wires (G&H wire company, Franklin, IN46131, USA) were used. The wires were cut into pieces of 5 centimeters in length and randomly divided into two groups of coated and uncoated (*n* = 50). Each group was further divided into four groups and subjected to either of the following tests: (I) tensile strength (*n* = 15), (II) three-point bending (*n* = 15), and (III) frictional resistance at 0° (*n* = 10) and 10° (*n* = 10). Sample size was calculated based on a standard deviation of SD = 1, a confidence level of 1. *α* = 0.95, and a precision of *d* = 0.25.

### 2.1. Preparation of Wires

In order to clean the surface of the oxidized layer and to improve the coated layer, the wires were placed in hydrochloride 0.5 Molar for 3 minutes, and the operator was told not to touch the wires with bare hands [19].

### 2.2. Coating with PVD

PVD coating was conducted in the Electrical and Computer Engineering School, Shiraz University. PVD with thermal evaporation (1 KW SCR stack & driver model 3AM, Edwards, England) was used to deposit thin films of Zn on the SS orthodontic wire. In PVD, heat was produced *via* thin sheet filaments of appropriate high-temperature metals, such as tungsten. The extremely high temperature with the vacuum chamber results in vaporized Zn. In this way, Zn traveled toward the chamber and hit its substrate, which was SS wire. The coating process was performed by holding one side of the wire and rotating it. The procedure was performed in 250°C for 4-5 h [20]. In total, 50 pieces were coated.

### 2.3. Observation and Measurement of the Zn Layer Surface

Specimens were examined by using a scanning electron microscope (SEM; TESCAN-Vega 3; TESCAN; Czech Republic) with 300x to 21,000x magnification to determine the coating layer morphology. In addition, the thickness of the coated layer was measured using SEM. The surface roughness (Ra) was measured, by Atomic Forced Microscopy (AFM; Full plus, Ara Pajooh, Iran).

Prior to the coating procedure, a plastic tape was placed on the middle part of the wire to hinder the Zn transudation beneath the taped area. The wires were placed in the PVD machine. After the coating procedure, the plastics were removed. The wires were observed with backscatter waves of SEM to determine the height difference between the coated and uncoated area. The difference was measured under the microscope.

**Figure 1:**
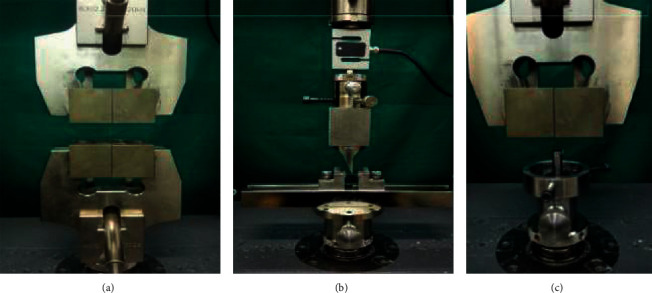
(a) Tensile strength, (b) three-point bending strength, and (c) friction forces of the wires were estimated using the universal testing machine.

Coated and uncoated specimens were further divided into four groups and subjected to the following tests: (I) tensile strength, (II) three-point bending, and (III) frictional resistance at 0° and 10°. All the mechanical tests were performed by the same operator, using the universal testing machine (Zwick Roell, Z020, Germany) (Figure 1).

### 2.4. Tensile Strength

The tensile test was performed, using the universal testing machine (Zwick Roell, Z020, Germany) with a load cell. The span of the wire placed between the grips was set at 20 mm [18]. SS wires were stretched until they snapped. The crosshead speed was 2 mm/min [11]. The proof strength was determined from the stress-strain diagram at a 0.2% strain. A total of 15 pieces were tested for the tensile strength test.

### 2.5. Three-point Bending

The flexural properties of the wires were estimated with the three-point bending test, using the universal testing machine (Zwick Roell, Z020, Germany). In total, 15 coated and uncoated wires were tested. The span length between fulcrums was 20 mm. Fulcrums were made of upper premolar standard MBT brackets (American Orthodontics, Sheboygan, USA). Brackets were fixed with cyanoacrylate adhesive. Wires were ligated to brackets by elastomeric modules (American Orthodontics, Sheboygan, USA). A crosshead was placed on the center of the specimen and engaged to move downward with 500 N loading at a rate of 6 mm/min. This caused the specimen bending to a final displacement of 3.1 mm under the applied load. Then, the crosshead was unloaded until reaching its original position. Bending force during loading was determined from the force-deflection diagram by recording the force readings that were taken at deflections of 1.0, 2.0, and 3.0 mm. The test was carried out at 37 ± 1°C temperature [18].

### 2.6. Friction Test

For the friction test, wires were ligated to a bracket with elastomeric modules (AO, U.S.A.). For this test, upper premolar standard MBT brackets (American Orthodontics, Washington, USA) were used. Each bracket was bonded to a metal surface with cyanoacrylate adhesive and angulated into 0 and 10 degrees. Friction forces of the wires were estimated with the universal testing machine (Zwick Roell, Z020, Germany). The upper end of each wire was attached to the tension load cell and pulled upward 5.0 mm at a speed of 0.5 mm/s with a free end. The span of the wire between the grip and bracket center was the standard 20 mm. The friction force values were from 0.5 s after starting of the measurement [18]. The bracket was replaced after sliding of each sample so that the conditions would be similar for all samples. In each angulation, ten coated and 10 noncoated wires were used.

### 2.7. Data Analysis

Data were analyzed using SPSS version 25 (SPSS Inc., Chicago, IL, USA). Mean and standard deviation were used to report the data. Normality was tested using the Kolmogorov–Smirnov test. An independent *t*-test was used to analyze the tensile strength data. the multivariate ANOVA test was used for frictional resistance, and repeated measurement ANOVA was applied for the three-point strength test. A significance level of *P* < 0.05 was considered for all analyses.

## 3. Results

**Figure 2:**
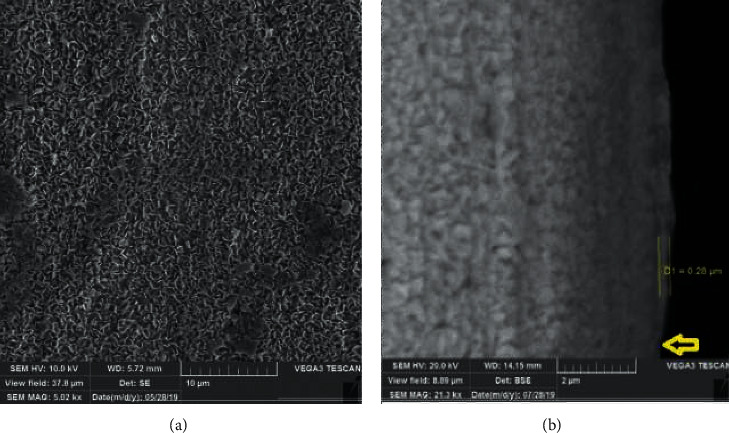
(a) The Zn-coated SS orthodontic wires. The uniform distribution of Zn on the wire can be observed (magnification: 5.02kx). (b) Backscatter waves of SEM showed the thickness of Zn on the SS orthodontic wires. This is due to the difference in electron reflection. The mean thickness of Zn was 0.28 *µ*m. There was no Zn layer on the bottom of the wire because it was covered with a plastic tape (magnification: 21.3kx).

The wires were observed by SEM. The Zn particles were homogenously spread all over the wire (Figure 2(a)). The backscatter waves were used to identify the Zn layer, and the mean Zn thickness was measured at 0.28 ± 0.006 *µ*m (Figure 2(b)).

**Figure 3:**
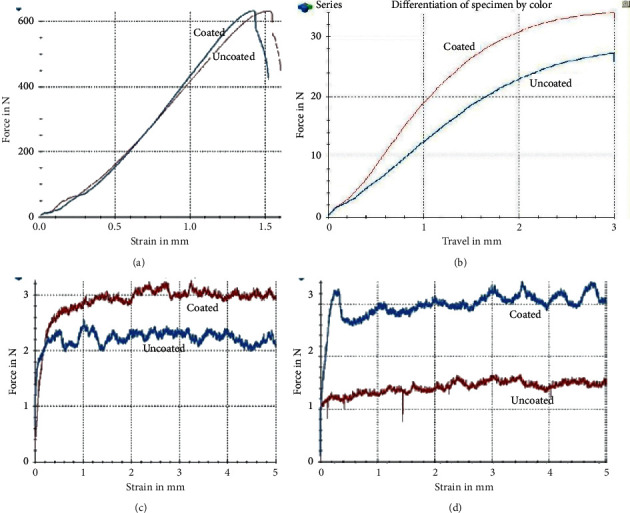
(a) The curve of stress-strain for modulus of elasticity in coated and uncoated wires. (b) Differences of load deflection for coated and uncoated wires. (c) The friction resistance (*N*) for coated and uncoated wires are compared at 0° (a) and 10° (b) angulations.

The curve of stress-strain for modulus of elasticity in coated and uncoated wires is shown in Figure 3(a). Mean values of modulus of elasticity for coated and uncoated wires were 637.93 ± 6.31 N and 629.00 ± 7.06 N, respectively. The coated wires had significantly higher strength than the uncoated ones (*P*=0.001).

**Figure 4:**
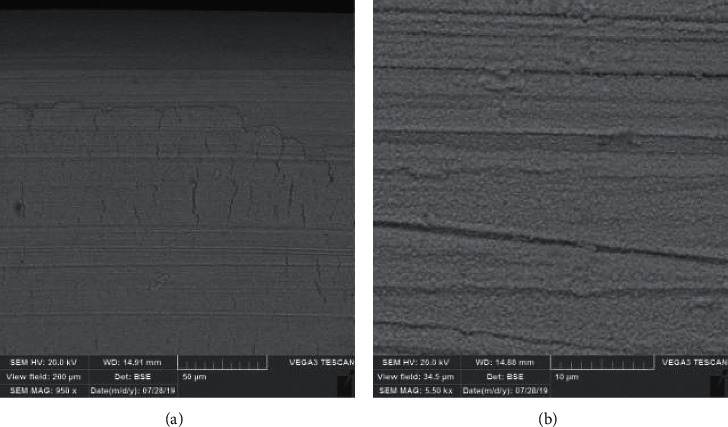
(a) The cracks appeared after the three-point bending test was performed (magnification: 950x). (b) The Zn layer is shown under the SEM after the friction resistance test was performed (magnification: 5.50kx).

Mean loading forces at the deflections of 1.0, 2.0, and 3.0 mm in the uncoated wires were 12.37 ± 0.43 N, 22.70 ± 0.67 N, and 27.02 ± 0.44 N, respectively (Table 1). The load-deflection curve shows the differences between coated and uncoated wires (Figure 3(b)). In the coated wires, mean loading forces were 18.75 ± 0.32 N, 32.72 ± 7.76 N, and 33.92 ± 0.42 N at the deflections of 3.0, 2.0, 1.0, and 0.5 mm, respectively. There was a significant difference between coated and uncoated wires in terms of three-point bending strength (*p* < 0.001).  Figure 4(a) revealed the surface layer after the three-point bending test under the SEM.

Figure 4(b) depicts the coated wire under the SEM after the friction test. The scratches can be seen clearly on the coated layer. The results of the friction test are shown in Table 2. In general, the presence or absence of particles on the wires showed significant differences in friction resistance (*p* < 0.001). However, the angle between the wire and bracket had no significant effect on friction (*p* > 0.05). The minimum and maximum frictional force were recorded at 0° in the coated wires (1.26 N) and at 10° in the uncoated wires (3.74 N).

The mean value of friction resistance between wires and brackets at 0° was 2.98 ± 0.54 N in the uncoated wires and 2.03 ± 0.43 N in the coated wires. The mean value of friction resistance between wires and brackets at 10° was 3.51 ± 0.81 N in the uncoated wires and 1.72 ± 0.21 N in the coated wires. The curve in Figure 3(c) shows the differences between coated and uncoated wires in each angulation. In each angle, between wires and brackets, there was a significant difference between coated and uncoated specimens (*p*=0.001 for 0° and *p* ≤ 0.001 for 10°).

In the coated wires, there was a minor reduction in frictional forces with an increase in the angle between the bracket and wire. The mean frictional forces were 2.03 ± 0.43 N and 1.72 ± 0.21 N at 0° and 10° angels, respectively (*p*=0.10). In the uncoated wires, a minor increase in friction between the bracket and wire was observed with an increase in the angle. The mean frictional forces at 0° and 10° angles were 2.98 ± 0.54 N and 3.51 ± 0.81 N (*p*=0.12).

## 4. Discussion

To the best of our knowledge, this study is the first to investigate the effect of Zn coating on SS orthodontic wires, using the PVD machine. The null hypothesis was rejected as the findings revealed that Zn coating was able to improve the tensile and load-bending strength and reduce the frictional resistance of SS orthodontic wires.

In the current study, 0.019 × 0.025 straight rectangular SS orthodontic wires were used due to their high stiffness, environmental stability, and low cost [2]. Furthermore, the 0.019 × 0.025 SS orthodontic wires are used in the “space closure stage” in which wires are preserved in the mouth for a long time. Even though a few studies had used SS wires for their coating procedure [15, 16, 18, 21–23], others used NiTi wires to improve the wire's mechanical properties [11]. However, it does not seem to be logical to use NiTi wires due to high costs and side effects, if kept in the mouth for a long time. Therefore, to inspect the effect of surface coating with Zn, SS orthodontic wires were implemented in the present study.

The alignment of teeth in orthodontic treatment is carried out by exploiting the mechanical properties of the used wire. As mentioned, SS wires are implemented in the space closure stage of orthodontic treatments as a beneficial aid for sliding mechanic. Thus, high breaking strength and stiffness is urgent for such SS wires [18]. Furthermore, to enhance treatment efficiency [5, 24] and oral health [6, 25], SS has to be improved with respect to its antibacterial activity and friction properties [4]. Previous research revealed that coating wires in this long-term stage might offer some advantages. Some studies have shown antibacterial effects following coating orthodontic wires with metals. Coating orthodontic wires with silver has been substantiated to enhance mechanical properties and reduce antibacterial activities [23]. Moreover, Zn has become increasingly popular for its inordinate antibacterial activity and its reduced friction resistance when it is used as coating on the surface of orthodontic wires [26].

In the present study, coated wires with Zn helped us to reach a perfect adherence and fill the wire's porosities. These alterations might lead to lower friction resistance in the coated wires. The reduced friction between wires and brackets is an important factor in orthodontics, since reduction in friction might help to reduce the treatment time and increase the anchorage control. This favorable effect can be achieved by wire coating as shown by Muguruma et al. [27]. Several studies have previously shown the effects of various coating applications on friction resistance. As reported by Usui et al., coating SS wires with “hard chrome carbide” reduced the friction resistance, but did not significantly improve other mechanical properties [21]. In another study by Zhang et al., using diamond-like carbon for coating SS wires was favored as it resulted in reduced friction resistance and modulus of elasticity as well as increased corrosion resistance [28]. In addition, Wei et al. coated SS orthodontic wires with a CNx film and detected significant reduction in the friction between wires and brackets, both in air and in artificial saliva. Moreover, Farronato et al. [29] reported that Teflon coating led to significant reduction in the sliding resistance of SS and NiTi wires. In line with the present study, Kachoei et al. [11] applied Zn on NiTi wires, using a chemical method. The authors proposed that Zn coating on NiTi wires could result in reduced friction resistance. In the present work, the PVD method was used to layer the 0.019 × 0.025 SS wires with Zn. As our findings revealed, a same reduction in the friction resistance of SS wires following the coating application was detected.

In the present research, to improve the mechanical properties, the wires were coated, using the PVD method. Thin-film coatings with PVD are a novel approach in orthodontics with promising features including an improved resistance to wear and corrosion, greater lubricity, reduced galling, and lengthy life span of the substrate. Furthermore, thin-film coatings are valuable in orthodontics since thicker coatings can lead to binding of the archwire to the bracket slot, increase the frictional resistance, and hinder the tooth movement. In addition, previous research verified that PVD-coated layers have the capability to endure bending stress and withstand prolonged clinical application in the oral cavity [20]. Moreover, since the PVD method is devoid of a need to reach a high temperature, this technique leads to easier layering and prevents the risk of burst.

Krishnan et al. [20] coated *β*-titanium archwires with TiAlN, using the PVD method. The authors reported that the mean value for coating thickness was 6.56 *µ*m. Another study by Sugisawa et al. [18] followed the same coating method and showed that the thickness of TiN coating on SS wires was 0.57 *µ*m and on NiTi wires was 0.58 *µ*m. Our findings revealed a mean thickness of 0.28 *µ*m Zn on SS wires. In general, the major drawback of the existing coated wires is that they are more likely to crack during bending [30, 31]. This can be explained by the fact that as the diameter of wires increase, they are more likely to deliver lower and less consistent force in bending. However, it seems that using Zn-coated wires might reduce the risk of crack propagation on the surface of the wire and offer a superior bending force during orthodontic treatment.

According to the results, the tensile strength significantly increased after coating with Zn. This indicates an increase in the stiffness following coating SS wires with Zn by the PVD method. Therefore, Zn-coated SS is expected to be useful for the closure space stage of orthodontic treatment. Previous studies have reported that the effect of plating orthodontic wires with materials, such as TiN, on their properties can vary with respect to the types of wires used. As previous studies showed, TiN coating of SS wires can increase the modulus of elasticity and break down the elongation of these wires. However, TiN plating has showed to have no significant effect on NiTi wires [18]. Moreover, Kachoei et al. used a chemical method to coat the NiTi wires with Zn and found that there was a significant increase in tensile strength in coated NiTi wires [11].

In this study, the three-point strength test was further investigated in Zn-coated SS wires. The load-deflection property is considered as one of the integral factors defining the biological potential of tooth movement [32]. The present study demonstrated a statistically significant difference in the increase of load-bending flexural strength between coated and uncoated SS wires. In fact, coated wires showed significantly greater flexural strength compared to uncoated ones. The results were in accordance with the findings of Sugisawa et al. [18] who performed on TiN-coated SS wires. In their study, the layering was carried out with the PVD method. The bending force in the coated wires was significantly higher than in uncoated ones. However, our result was in contrast to that of Usui et al. [21] who coated SS wires with “hard chrome carbide.” The authors reported that this layer made no significant differences in flexural strength in comparison with the control group.

The findings of the present study approved the positive effects of Zn coating on enhancing the mechanical properties and reducing the friction force of SS orthodontic wires. One of the limitations of our research was that the clinical conditions could not be precisely simulated in this *in vitro* study. If verified by clinical studies, these findings can be of great assistance in practice by providing superior anchorage control and diminishing the risk of root resorption. Therefore, the authors suggest further clinical studies on this topic. Moreover, it should be taken into account that wear [33] or brushing [34] can alter the surface characteristics of the orthodontic materials. Yet, the current study did not consider the effect of brushing or long-term wear on the coated stainless steel wires. Therefore, further studies are needed in order to also consider the possible effects of other unexplored variables.

## 5. Conclusions

Within the limitations of this study, it was concluded that Zn coating by the PVD method might improve tensile and load-bending strength of SS orthodontic wires. Zn coating can also reduce the frictional resistance which is applicable in tooth movement with sliding mechanics.

## Figures and Tables

**Table 1:** Mean ± SD of three-point bending strength values (*N*) in coated and uncoated wires.

Groups	Coated	Uncoated	
1 mm	18.75 ± 0.32^b,A^	12.37 ± 0.43^c,B^	*P* < 0.001
2 mm	32.72 ± 7.76^a,A^	22.70 ± 0.67^b,B^	
3 mm	33.92 ± 0.42^a,A^	27.02 ± 0.44^a,B^	
*P* value	*P* < 0.001	*P* < 0.001	

Different lower-case letters show the significant difference in a column (between different deflections). Different upper-case letters show the significant difference in a row (between coated and uncoated).

**Table 2:** Mean ± SD of friction resistance values (*N*) in coated and uncoated wires.

Groups	Coated	Uncoated	*P* value
0°	2.03 ± 0.43	2.98 ± 0.54	0.001
10°	1.72 ± 0.21	3.51 ± 0.81	<0.0001
*P* value	0.10	0.12	—

## Data Availability

The data used to support the findings of this study are included within the article.

## References

[1] Sfondrini M. F., Cacciafesta V., Maffia E. (2009). Chromium release from new stainless steel, recycled and nickel-free orthodontic brackets. *The Angle Orthodontist*.

[2] Kapila S., Sachdeva R., Orthopedics D. (1989). Mechanical properties and clinical applications of orthodontic wires. *American Journal of Orthodontics and Dentofacial Orthopedics*.

[3] Isak J., Mahedira S., Chandrashekar B., Reddy V., Ramesh K. P., Shetty B. (2016). Effects of clinical recycling on mechanical properties of three commonly used types of orthodontic archwires. *Saudi Journal of Oral and Dental Research*.

[4] Oh K.-T., Kim K.-N. (2005). Ion release and cytotoxicity of stainless steel wires. *European Journal of Orthodontics*.

[5] Muguruma T., Iijima M., Brantley W. A., Nakagaki S., Endo K., Mizoguchi I. (2011). Frictional and mechanical properties of diamond-like carbon-coated orthodontic brackets. *The European Journal of Orthodontics*.

[6] Peng L., Chang L., Liu X. (2017). Antibacterial property of a polyethylene glycol-grafted dental material. *ACS Applied Materials & Interfaces*.

[7] Rosenbloom R. G., Tinanoff N. (1991). Salivary Streptococcus mutans levels in patients before, during, and after orthodontic treatment. *American Journal of Orthodontics and Dentofacial Orthopedics*.

[8] Scheie A. A., Arneberg P., Krogstad O. (1984). Effect of orthodontic treatment on prevalence of Streptococcus mutans in plaque and saliva. *European Journal of Oral Sciences*.

[9] Featherstone J. D. (2003). The caries balance: contributing factors and early detection. *Journal of the California Dental Association*.

[10] Dhas S. P., Shiny P. J., Khan S., Mukherjee A., Chandrasekaran N. (2014). Toxic behavior of silver and zinc oxide nanoparticles on environmental microorganisms. *Journal of Basic Microbiology*.

[11] Kachoei M., Nourian A., Divband B., Kachoei Z., Shirazi S. (2016). Zinc-oxide nanocoating for improvement of the antibacterial and frictional behavior of nickel-titanium alloy. *Nanomedicine*.

[12] Mohammed N. R., Mneimne M., Hill R. G., Al-Jawad M., Lynch R. J. M., Anderson P. (2014). Physical chemical effects of zinc on in vitro enamel demineralization. *Journal of Dentistry*.

[13] Lynch R. J. M., Churchley D., Butler A. (2011). Effects of zinc and fluoride on the remineralisation of artificial carious lesions under simulated plaque fluid conditions. *Caries Research*.

[14] Lippert F. (2012). Dose-response effects of zinc and fluoride on caries lesion remineralization. *Caries Research*.

[15] Kachoei M., Eskandarinejad F., Divband B., Khatamian M. (2013). The effect of zinc oxide nanoparticles deposition for friction reduction on orthodontic wires. *Dental Research Journal*.

[16] Behroozian A., Kachoei M., Khatamian M., Divband B. (2016). The effect of ZnO nanoparticle coating on the frictional resistance between orthodontic wires and ceramic brackets. *Journal of Dental Research, Dental Clinics, Dental Prospects*.

[17] Shah P. K., Sharma P., Goje S. K. (2018). Comparative evaluation of frictional resistance of silver-coated stainless steel wires with uncoated stainless steel wires: an in vitro study. *Contemporary Clinical Dentistry*.

[18] Sugisawa H., Kitaura H., Ueda K. (2018). Corrosion resistance and mechanical properties of titanium nitride plating on orthodontic wires. *Dental Materials Journal*.

[19] Oguike R. S. (2014). Corrosion studies on stainless steel (FE6956) in hydrochloric acid solution. *Advances in Materials Physics and Chemistry*.

[20] Krishnan V., Krishnan A., Remya R. (2011). Development and evaluation of two PVD-coated *β*-titanium orthodontic archwires for fluoride-induced corrosion protection. *Acta Biomaterialia*.

[21] Usui T., Iwata T., Miyake S. (2018). Mechanical and frictional properties of aesthetic orthodontic wires obtained by hard chrome carbide plating. *Journal of Dental Sciences*.

[22] Gracco A., Dandrea M., Deflorian F. (2019). Application of a molybdenum and tungsten disulfide coating to improve tribological properties of orthodontic archwires. *Nanomaterials*.

[23] Mhaske A. R., Shetty P. C., Bhat N. S. (2015). Antiadherent and antibacterial properties of stainless steel and NiTi orthodontic wires coated with silver against Lactobacillus acidophilus—an in vitro study. *Progress in Orthodontics*.

[24] Burrow S. J., Orthopedics D. (2009). Friction and resistance to sliding in orthodontics: a critical review. *American Journal of Orthodontics and Dentofacial Orthopedics*.

[25] Cao B., Wang Y., Li N., Liu B., Zhang Y. (2013). Preparation of an orthodontic bracket coated with an nitrogen-doped TiO_2_-xNy thin film and examination of its antimicrobial performance. *Dental Materials Journal*.

[26] Ramazanzadeh B., Jahanbin A., Yaghoubi M. (2015). Comparison of antibacterial effects of ZnO and CuO nanoparticles coated brackets against Streptococcus mutans. *Journal of Dentistry (Shiraz, Iran)*.

[27] Muguruma T., Iijima M., Brantley W. A., Mizoguchi I. (2011). Effects of a diamond-like carbon coating on the frictional properties of orthodontic wires. *The Angle Orthodontist*.

[28] Zhang H., Guo S., Wang D., Zhou T., Wang L., Ma J. (2016). Effects of nanostructured, diamond like, carbon coating and nitro carburizing on the frictional properties and biocompatibility of orthodontic stainless steel wires. *The Angle Orthodontist*.

[29] Farronato G., Maijer R., Carìa M. P., Esposito L., Alberzoni D., Cacciatore G. (2011). The effect of teflon coating on the resistance to sliding of orthodontic archwires. *The European Journal of Orthodontics*.

[30] Bradley T. G., Berzins D. W., Valeri N., Pruszynski J., Eliades T., Katsaros C. (2013). An investigation into the mechanical and aesthetic properties of new generation coated nickel-titanium wires in the as-received state and after clinical use. *European Journal of Orthodontics*.

[31] Washington B., Evans C. A., Viana G., Bedran-Russo A., Megremis S. (2015). Contemporary esthetic nickel-titanium wires: do they deliver the same forces?. *The Angle Prthodontist*.

[32] Elayyan F., Silikas N., Bearn D. (2008). Ex vivo surface and mechanical properties of coated orthodontic archwires. *The European Journal of Orthodontics*.

[33] Sartawi S., Salim N. A., Taim D. (2020). Awareness and treatment decisions on tooth wear among Jordanian dentists and prosthodontists: a cross-sectional survey study. *International Journal of Dentistry*.

[34] Scribante A., Vallittu P., Lassila L. V. J. (2019). Effect of long-term brushing on deflection, maximum load, and wear of stainless steel wires and conventional and spot bonded fiber-reinforced composites. *International Journal of Molecular Sciences*.

